# Subcostal Echocardiographic Imaging in Neonatal and Pediatric Intensive Care

**DOI:** 10.3389/fped.2021.471558

**Published:** 2021-06-24

**Authors:** Stefan Kurath-Koller, Martin Koestenberger, Georg Hansmann, Massimiliano Cantinotti, Cecille Tissot, Hannes Sallmon

**Affiliations:** ^1^Division of Pediatric Cardiology, Department of Pediatrics, Medical University Graz, Graz, Austria; ^2^Department of Pediatric Cardiology and Critical Care, Hannover Medical School, Hanover, Germany; ^3^Fondazione Consiglio Nazionale delle Ricerche Area (CNR)-Regione Toscana G. Monasterio (FTGM), Pisa, Italy; ^4^Center de Pediatrie, Clinique des Grangettes, Chêne-Bougeries, Switzerland; ^5^Department of Pediatric Cardiology, Charité–Universitätsmedizin Berlin, Berlin, Germany

**Keywords:** echocardiography, intensive & critical care, neonatal intensive care, subcostal approach, emergency & critical care

## Introduction

Point of care ultrasound is essential in any emergency department (ED) and intensive care unit (ICU) and represents a well-established procedure in ICUs and EDs all over the world. In terms of cardiac evaluation, focused cardiac ultrasound (FOCUS) is recommended by a consensus statement of the American Society of Echocardiography (ASE) and the American College of Emergency Physicians (ACEP), outlining the important role of FOCUS in clinical medicine (1). The principal role for FOCUS is the time-sensitive hemodynamic assessment of the acutely ill patient (2, 3).

Neonatologists and pediatric intensivists may benefit from using FOCUS to assess a variety of objectives (e.g., guiding therapy or assess pericardial effusion) (4, 5). In any case, obtaining clearly defined echocardiographic views is required in order to perform adequate assessment. In expert hands, subcostal area views constitute an easily accessible option to perform a structured echocardiographic evaluation of the heart with the advantage of achieving clear views quickly, especially in neonates and infants, in whom cardiac structures are in proximity to the echocardiography probe (4). Herein, we provide a literature review of the current state of subcostal imaging supported by technical information on how to acquire subcostal window views.

## Definition of Focused Cardiac Ultrasound Assessment

FOCUS (also termed point-of-care cardiac ultrasound or bedside cardiac ultrasound, among others) represents a focused examination of the cardiovascular system, performed by a physician who uses ultrasound as an adjunct to the physical examination. The aim is to recognize specific sonographic signs that represent a narrow list of potential diagnoses in specific clinical settings (6).

## Goals of Focus Performed by Non-Specialists

FOCUS can be a helpful tool in the hands of trained non-specialists (i.e., pediatric intensive care specialists, neonatologists, or pediatric emergency physicians). Its goal is the early identification of limited hemodynamic changes, thus expediting establishment of initial diagnoses and clinical decision making (7). Trained non-specialists should be able to asses and focus on left ventricular systolic function, volume status/response to fluid resuscitation, pericardial effusion/cardiac tamponade, and right ventricular systolic function and pulmonary hypertension (7–10). By incorporating a few prechosen echocardiographic views, several conditions of interest can easily be confirmed or ruled out by targeted FOCUS examination (Table 1). Subsequent referral for a comprehensive echocardiographic study should be considered to delineate and quantify all findings, including incidental findings, which may unrecognized during FOCUS (7). Throughout FOCUS, abnormal findings are classified as present or absent using a predefined specific imaging protocol. The performing physician must adhere to this predefined protocol in order to provide specific diagnosis in a timely fashion while avoiding exaggerated examination. This approach allows for the rapid bedside detection of some selected pathologic conditions, which then must be interpreted in concert with all physical findings and the patient's history.

**Table 1:** Differential diagnoses to be assessed using FOCUS.

**Differential Diagnosis**	**Assessment by FOCUS**
Pericardial effusion	Pericardial fluid from subcostal window, apical, and parasternal views
Left ventricular function	“Eyeballing” of LV function from subcostal window views, estimation of LV function from apical, and parasternal view
Right ventricular function	“Eyeballing” of RV function, RV dimensions and S-TAPSE from subcostal window views; TAPSE from apical view
Pulmonary hypertension	S-TAPSE and RV dimensions from subcostal window views; TAPSE, PAAT, or systolic PAP from apical and parasternal views

*LV, left ventricle; RV, right ventricle; S-TAPSE, subcostal tricuspid annular plane systolic excursion; TAPSE, tricuspid annular plane systolic excursion; PAAT, pulmonary artery acceleration time; PAP, pulmonary artery pressure*.

## Global Cardiac Systolic Function

FOCUS aims to support and guide clinical decision-making. For example, in a patient presenting with acute shortness of breath or palpitations, FOCUS may detect impaired systolic contractility, thereby aiding decision making regarding pharmacological or interventional treatment (11).

Subcostal area views may allow for estimation of LV and RV function. This can be achieved using eyeballing of LV contraction in the subcostal short axis view, RV dimension measurement, or S-TAPSE determination (12, 13). However, except for RV dimension estimation and S-TAPSE, currently no normative values regarding left and right ventricular function parameters (e.g., ejection fraction, aortic, or pulmonary VTI) are available in the pediatric population.

## Pericardial Effusion and Cardiac Tamponade

Pericardial effusion (PE) resulting in cardiac tamponade is a critical condition which may be easily diagnosed using FOCUS. Pericardial tamponade is a clinical diagnosis supported by an echocardiographic view of pericardial fluid/blood in addition to clinical signs (e.g., arterial hypotension, tachycardia, or distended neck veins) (1). FOCUS will reveal PE with early diastolic RV collapse (1, 14, 15) and is highly accurate in diagnosing PE (sensitivity 96%, specificity 98%) (1, 15). For life-saving pericardiocentesis ultrasound may be used to guide needle insertion resulting in less complication and higher success rates, when compared to intervention without ultrasound based guidance (1, 14, 16). Subcostal area views may provide a quick assessment of a pericardial effusion and support guidance to needle insertion during pericardiocentesis. Furthermore, impaired RV function may be quantified by determination of S-TAPSE and RV dimensions (12, 13).

## Right Ventricular Function

Evaluation of RV function often is crucial to estimate disease severity when treating children suffering from congenital heart disease (CHD) (2, 3). RV dimensions may be assessed using different projections (apical four chamber/five chamber view, parasternal long/short axis), and according to adult recommendations measurements of both, linear dimensions and areas should be achieved for proper functional evaluation of the RV (5, 17, 18). Recently, Cantinotti et al. (12) published nomograms for echocardiographic RV subcostal view dimensions in children. Estimation of RV function from subcostal area views may be performed using RV dimension measurements as well as S-TAPSE as a function of RV systolic shortening (13).

## Pulmonary Hypertension and Pulmonary Hypertensive Crisis

For echocardiographic evaluation and confirmation of PH the following parameters should be applied: estimation of systolic pulmonary artery pressure (PAP) by estimation of RV systolic pressure (RVSP) using the maximum velocity of the tricuspid regurgitation (TR) flow by continuous wave (CW)-Doppler; mean PAP and end-diastolic PAP by CW-Doppler velocity of pulmonary regurgitation (PR) flow; determination of RV longitudinal systolic function by tricuspid annular plane systolic excursion (TAPSE); RV strain and strain rate measurements; 3D echocardiographic RV volume determination; RV systolic to diastolic duration ratio; tissue Doppler velocities; RV/LV diameter ratio; eccentricity index, PA acceleration time (PAAT) (19).

Subcostal area views may allow an estimation of PH using S-TAPSE, tricuspid, and pulmonary valve regurgitation and RV dimensions (12, 13, 20). However, as sufficient literature supporting evaluation of PH from subcostal area views is lacking, assessment of PH should not be performed from subcostal area views alone, but by comprehensive echocardiographic evaluation as recently described (20).

## Persistent Pulmonary Hypertension of the Newborn

Acute PH in newborn patients (persistent pulmonary hypertension of the newborn–PPHN, or persistent fetal circulation–PFC), is associated with high mortality despite ongoing advances in neonatal medicine. These infants are typically very sick and benefit from time-sensitive optimization of cardiorespiratory support. Clinical presentation may be misleading and mimic cyanotic congenital heart disease. In this case, FOCUS enables to rule out underlying major CHD, diagnose PH and assess its severity. Furthermore, it allows for efficacy monitoring of specific therapies (21, 22).

By using subcostal area views an estimation of PH may be possible using RV dimension measurements, assessment of shunt direction *via* foramen ovale (often bidirectional in the case of PPHN), TR jet velocity (used to estimate RVSP), S-TAPSE (determination of systolic RV function), or PR jet velocity (used to estimate mPAP and dPAP) (12, 13). However, as previously mentioned, assessment of PH should not be made from subcostal area views alone but requires a comprehensive echocardiographic evaluation (20). Subcostal area views may, however, be used as a last resort in situations of severe over-inflation, pneumothorax, following sternotomy, or others.

Following initial management, however, all patients with abnormal findings not previously documented should be referred to a comprehensive echocardiographic examination by a pediatric cardiologist. FOCUS performed by non-experts is not intended to completely evaluate a symptomatic cardiac patient.

Recently, FOCUS was demonstrated to be of use in assessment of pediatric septic shock, determining fluid responsiveness, obstructive physiology, and myocardial dysfunction (23).

FOCUS is both, sensitive and specific for identifying pericardial effusion and left ventricular systolic dysfunction when performed by non-experts with appropriate training (24). The latter, of course, is of major importance and needs to be updated regularly on a structured basis (25, 26).

## Advantages of Subcostal Imaging

General advantages of cardiac ultrasound, e.g., being a noninvasive risk-free method, performed serially and in real time, do of course also apply to FOCUS (7). Using the subcostal window views provides good spatial appreciation of cardiac structures, especially in congenital heart disease. Furthermore, it may provide improved imaging quality when transthoracic imaging is suboptimal, e.g., with pneumothorax or in infants after cardiac surgery with bandages in place. Especially in small children, toddlers and newborn/premature infants subcostal window views provide quick access and good imaging quality for FOCUS. Subcostal window views allow excellent assessment of pericardial effusions, left- and right ventricular function, venous filling, and volume status. Exploring the subcostal window views, one may assess the majority of cardiac anatomy, with restricted access to the great vessels (except for their most proximal part).

## Disadvantages of Subcostal Imaging

Due to spatial conditions, subcostal window views are less useful in adolescents, especially when obese. Even though transthoracic imaging can also be difficult in this particular patient group, it seems superior in image acquisition and quality when compared to subcostal window views. In the postoperative infant with mediastinal and pericardial drainages in place, usually placed in the subxiphoid region, obtaining subcostal window views can be challenging. A clear disadvantage of the subcostal approach lies within the assessment of the great arteries. While the proximal parts including the aortic and pulmonary valve are properly assessable, spatial distance to the ultrasound probe interferes with the assessment of more distal parts, including the aortic arch and pulmonary artery branches. These limitations also exist for assessment of persistent ductus arteriosus (PDA), one major issue in neonatal intensive care. Especially, hemodynamic significance of a PDA should not be assessed from subcostal area views as adequate visualization of the aortic isthmus is rarely possible to rule out coarctation and/or interrupted aortic arch.

## Current Literature and Practice Guidelines Incorporating Subcostal Window Views

Guidelines on FOCUS in pediatric patients were published by the American Society of Echocardiography in 2013 (6). In 2014, the American Society of Echocardiography also published evidence-based recommendations for FOCUS (26). Several articles report on the use of FOCUS in pediatric emergency care (27), pediatric critical care (28), and neonatology (29). However, the subcostal window views are alluded but remain to be described in detail by current publications. The majority of reports on subcostal window views originate from the 80s of the 20th century, reporting assessment of atrial septum defects, transposition of great arteries, or right ventricular wall thickness (28, 29).

More recent literature provides normative values and z-scores on right ventricular structure and function, including end diastolic basal-apical and latero-lateral diameters, end diastolic and end systolic area, four chamber end diastolic and end systolic area and length, end diastolic basal (RV1) and mid-cavity (RV2) diameters (12), as well as subcostal tricuspid annular plane systolic excursion (13).

To date, no true comparative studies outlining the advantages and disadvantages of subcostal window views over transthoracic views exist. Future studies on this topic are warranted. Despite one study comparing 3-D echo from apical, parasternal, and subcostal view to display the four chambers of the heart found subcostal window views to be inferior as compared to the apical views (30), 3-D studies remain beyond the goals of FOCUS.

## Technical Information and Image Acquisition From Subcostal Window Views

Proper orientation of the acquired image on the screen is important (e.g., the LV should be positioned on the right side of the screen for subcostal views). Sweeps will provide detailed imaging and allow for assessment of the atrial and ventricular septum, atrioventricular valves, chambers, and systemic venous return. Furthermore, to assess right and left ventricular outflow tracts, rotating the transducer probe provides suitable imaging. Echocardiography is a skill dependent diagnostic tool and it is essential to outline that proper education and frequent training is required, even more so in infants and children. Therefore, “skill level” is of outmost importance and should be considered when reporting research results on FOCUS applications (31). Hébert et al. (32) emphasize the importance of expert training and need for competency based assessment.

There are some differences between the adult and pediatric population, e.g., which transducer to use in obtaining best views.

### Adults

Low-frequency phased array transducers are commonly employed in transthoracic echocardiography (TTE) (1, 14, 33–35). Low frequency probes focus at 12–16 cm. It may be necessary to have the patient inhale and hold their breath in order to obtain the short axis images.

### Neonates and Children

Using transducers with different frequencies is necessary in pediatric patients, ideally ranging from 12 to 4 MHz, as the patient population ranges from preterm infants to adolescents and young adults. Thereby imaging at different depths can be achieved. High frequency transducers come with high resolution, focusing at a depth of 4 to 5 cm, while lower frequency transducers focus at a greater depth and provide lower resolution. Therefore, using high frequency transducers in neonates and infants, in whom subcostal area views succeed best, is warranted (4).

In larger children and adolescents, similar to adults, it may be necessary to have the patient inhale and hold their breath in order to obtain the short axis images. However, in neonates and infants these views can be achieved quite easily and independently from respiration due to the proximity of the heart. In pediatric and neonatal intensive care patients, and in some children suffering from lung disease, as well as when the acoustic window is narrow and poor, the subcostal area approach may yield the best images of the heart (4).

Adequate knowledge and skill level can be acquired through courses on FOCUS, endorsed by national or international associations such as the AEPC (Association for European Pediatric and Congenital Cardiology) or AAP (American Academy of Pediatrics). Experts in the field of echocardiography are encouraged to teach FOCUS essentials to e.g., pediatric intensivists and neonatologists. In combination with a structured echocardiographic education and training, this should assure a high quality of FOCUS. Appropriate boundaries, e.g., when to consult an echocardiography expert and scope of competencies should be regulated by local echo-labs or pediatric cardiologists. However, assessments of global cardiac function and pericardial effusions may be adequately achieved by properly trained pediatric intensivists or neonatologists.

The following section provides an overview on specific subcostal window views.

### Subxiphoid Abdominal Short Axis View (Situs View)

**Figure 1:**
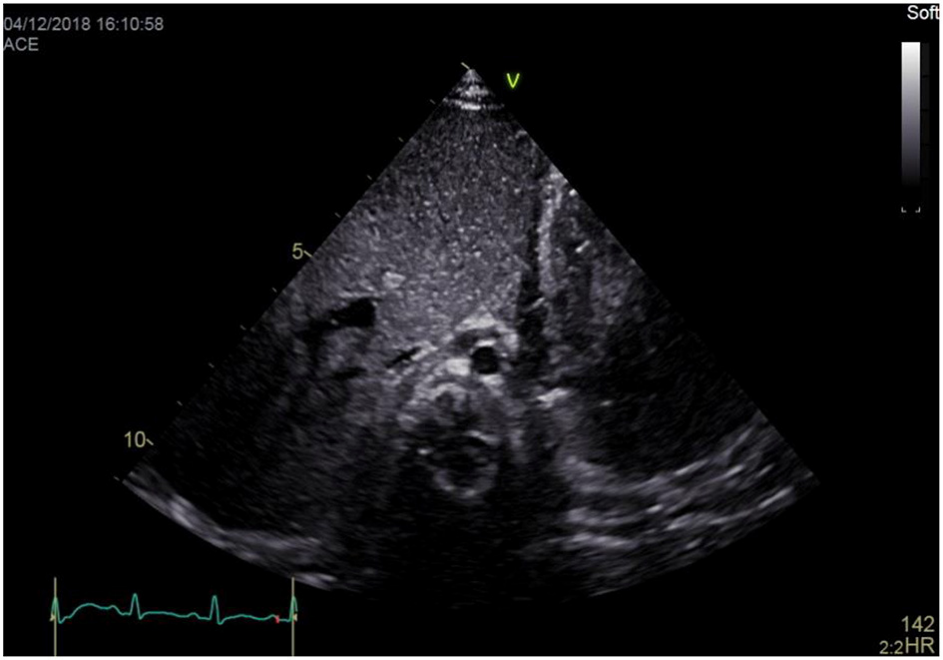
Subxiphoid abdominal short axis view (“Situs view”).

a) How to obtain (Figure 1):

Place the transducer just inferior to the xiphoid process, with the transducer dot pointing at 3 o'clock.

b) What to identify:

Inferior vena cava (IVC), abdominal aorta, spine, liver, both hemidiaphragms.

c) Interpretation:

This view shows the descending aorta to the left and the IVC to the right of the spine. Aortic flow should be visualized using color Doppler with a pulsating pattern in 2D echocardiography. The liver is displayed to the right. This enables determination of normal abdominal visceral situs or otherwise situs anomalies, and levocardia when the cardiac apex is located to the left. Both hemidiaphragms are seen, moving symmetrically with respiration. Moreover, focusing on the hemidiaphragms fluid in the pleural cavity (pleural effusions) may be identified.

### Subxiphoid Abdominal Long Axis View (IVC View)

**Figure 2:**
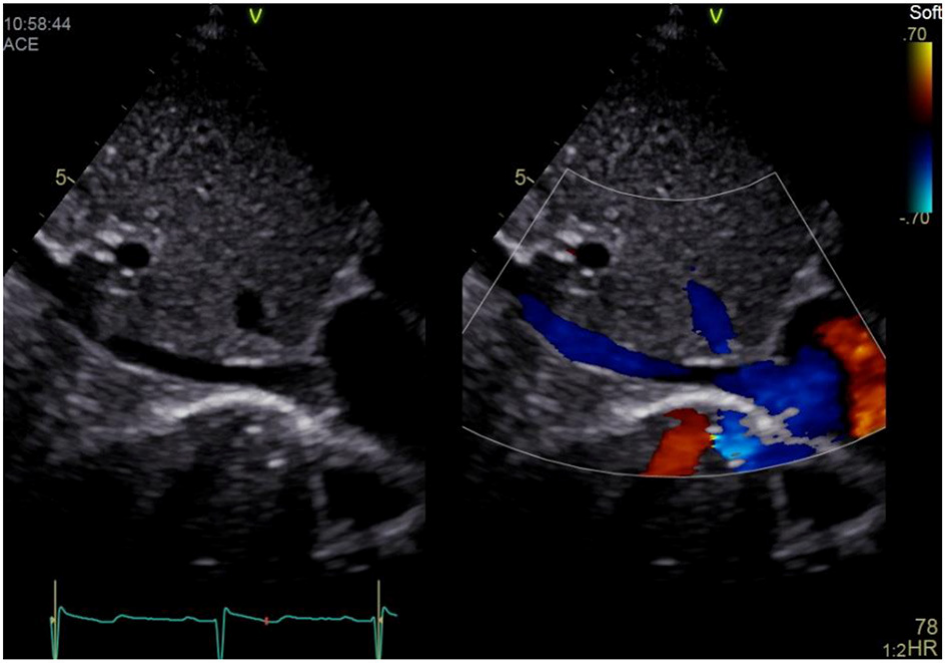
Subxiphoid abdominal long axis view (“IVC view”).

a) How to obtain (Figure 2):

Place the transducer below the xiphoid process and slide a bit to the patients' right. The transducer dot points at 1 o'clock. Slide until you get a clear view on the IVC running longitudinally by the liver, entering the right atrium (RA).

b) What to identify:

IVC, hepatic veins, RA, interatrial septum (IAS), left atrium (LA).

c)Interpretation:

IVC filling and collapsibility can be assessed (for details see below under “Volume Assessment”) and the venous flow pattern is displayed at the IVC-RA junction. Furthermore hepatic vein distension or reverse flow may be noted in case of elevated RA pressure, i.e., right heart failure or pulmonary hypertension (PH). In newborns and infants it is possible to display the IAS and the LA. A persistent foramen ovale (PFO) or atrial septal defect (ASD) may be seen. Using color Doppler determination of left-to-right or right-to-left shunt flow is possible.

### Sweep (Aorta)

**Figure 3:**
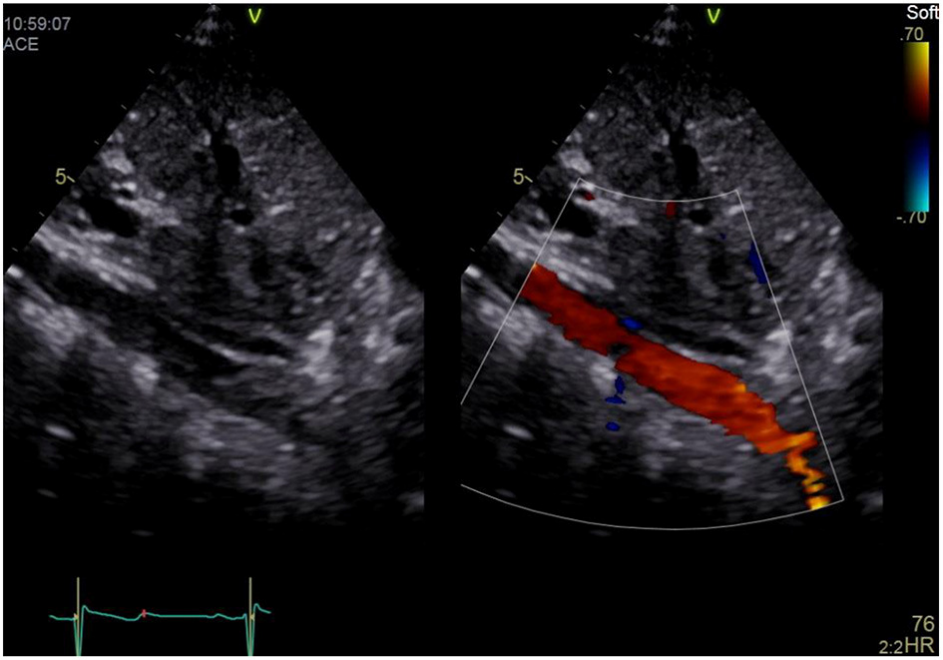
Subxiphoid short axis “Aortic Sweep”.

a) How to obtain (Figure 3):

Tilt the transducer to the patients' left until the abdominal aorta is displayed longitudinally.

b) What to identify:

Abdominal and descending aorta, superior mesenteric artery (SMA), RA.

c)Interpretation:

Using color Doppler the abdominal and descending aorta may be identified showing a pulsatile flow pattern. Diastolic flow may be seen in upstream narrowing of the aorta, e.g., coarctation. Reverse flow pattern may be seen in significant aortic regurgitation (AR) or persistent ductus arteriosus (PDA). Furthermore, pulsatile flow and Doppler tracings of the SMA allow for resistance index (RI) calculation and estimation of visceral organ perfusion (1).

### Subxiphoid Coronal Long Axis View

Place the transducer just inferior to the xyphoid process, positioning the index marker to the patient's left side and angling the transducer superiorly toward the left thorax. With the transducer dot pointing at 4 o'clock, tilting cranially for the subxiphoid four chamber view should be performed as described above. This represents the top view in subxiphoid long axis.

### Subxiphoid Coronal Long Axis Four Chamber View

**Figure 4:**
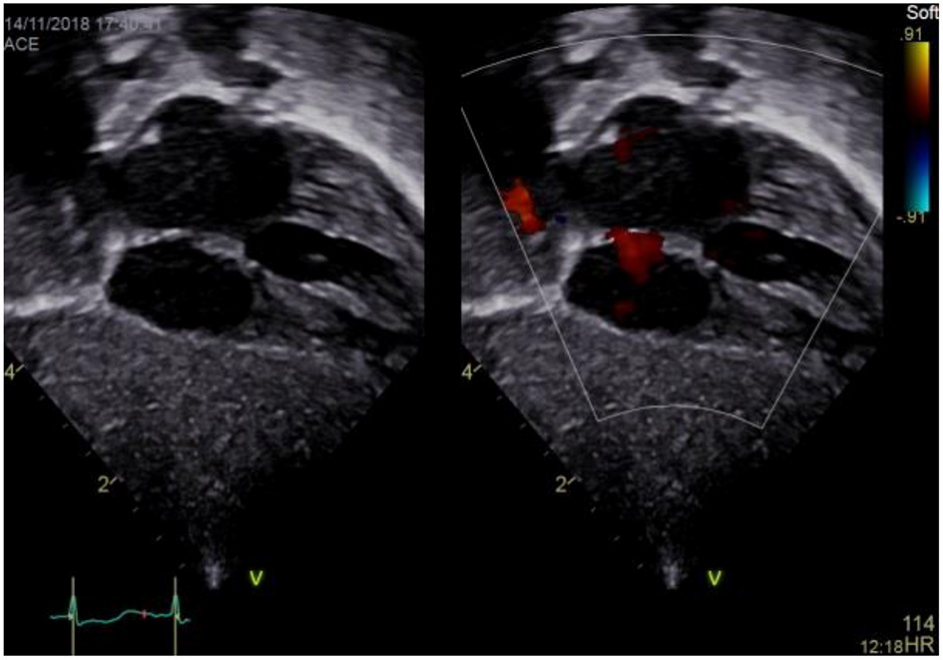
Subxiphoid four chamber view.

a) How to obtain (Figure 4):

Place the transducer just inferior to the xyphoid process, positioning the index marker to the patient's left side and angling the transducer superiorly toward the left thorax. Transducer dot pointing at 4 o'clock, tilting cranially.

b) What to identify:

RA, LA, right ventricle (RV), left ventricle (LV), IAS, interventricular septum (IVS), tricuspid valve (TV), mitral valve (MV), (left upper pulmonary vein LUPV).

c) Interpretation:

The subcostal four chamber view provides an overall view of the heart. It is one of the best views for detecting pericardial effusions and both atrial and ventricular septal defects because the jets usually arise relatively perpendicular to the septae and parallel to the ultrasound beam. Also, the subcostal four chamber view is one of the best views for assessing the functional status of an ASD closure device. Moreover, ventricular or atrial enlargement may be identified as well as tricuspid or mitral regurgitation. Estimation of left and right ventricular function using “eyeballing” is possible. LV or RV hypertrophy or dilation may be seen.

### Subxiphoid Coronal Long Axis–Coronary Sinus Sweep

**Figure 5:**
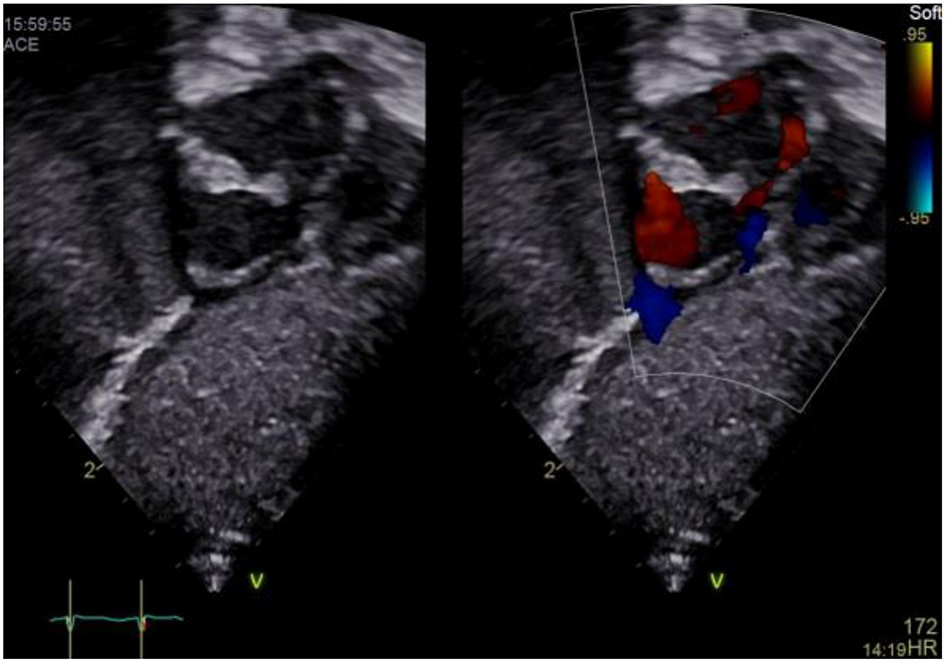
Subxiphoid long axis view–CS sweep.

a) How to obtain (Figure 5):

From the subxiphoid long axis four chamber view, as described above, tilt the transducer caudally until you visualize the coronary sinus (CS).

b) What to identify:

RA, CS, LA, RV, LV, IVS, IAS in part.

c) Interpretation:

Coronary sinus (CS) blood flow can be identified using color Doppler. Prominent CS flow may be seen in partial anomalous pulmonary venous return (PAPVR), total anomalous pulmonary venous return (TAPVR), CS ASD, persistent left superior vena cava (PLSVC) draining to the dilated CS, or high RA pressure as seen in some cases of PH (2). A CS ASD due to unroofing of the CS might be suspected by color Doppler visualizing of a left-to-right or right-to-left shunt flow between LA and CS. Again a VSD or ASD might be visualized.

### Subxyphoid Coronal Long Axis–LVOT Sweep

**Figure 6:**
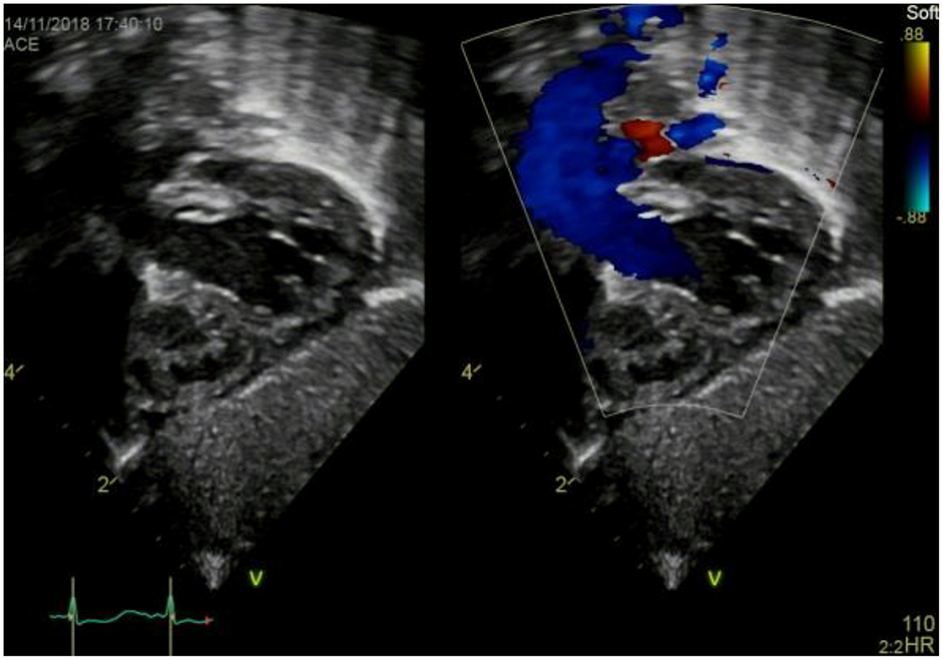
Subxiphoid “LVOT view”.

a) How to obtain (Figure 6):

From the subxiphoid four chamber view, tilt the transducer cranially until you visualize the left ventricular outflow tract (LVOT) and the aortic valve (AV).

b) What to identify:

SVC, RA, TV, RV, LV, AoV, ascending aorta, IVS, pulmonary artery (PA).

c) Interpretation:

The SVC entering the RA and associated flow can be visualized and SVC stenosis may be suspected if elevations of flow velocity are present. TV regurgitation may be visualized as well as a muscular or sub aortic VSD using color Doppler. A left ventricular outflow tract (LVOT) narrowing might be suspected by visualizing turbulences using color Doppler as well as obstructing tissue in 2D echocardiography. Aortic regurgitation (AR) may be seen using color Doppler, and additional LV hypertrophy may be suspected in 2D echocardiography. Using spectral Doppler, aortic velocity time integrals (AoVTI) may be measured and allow for serial monitoring of LV output (3). However, normative values for measurements from the subxiphoid area views currently do not exist for the pediatric population.

### Subxyphoid Coronal Long Axis–RVOT Sweep or “Right Heart View”

**Figure 7:**
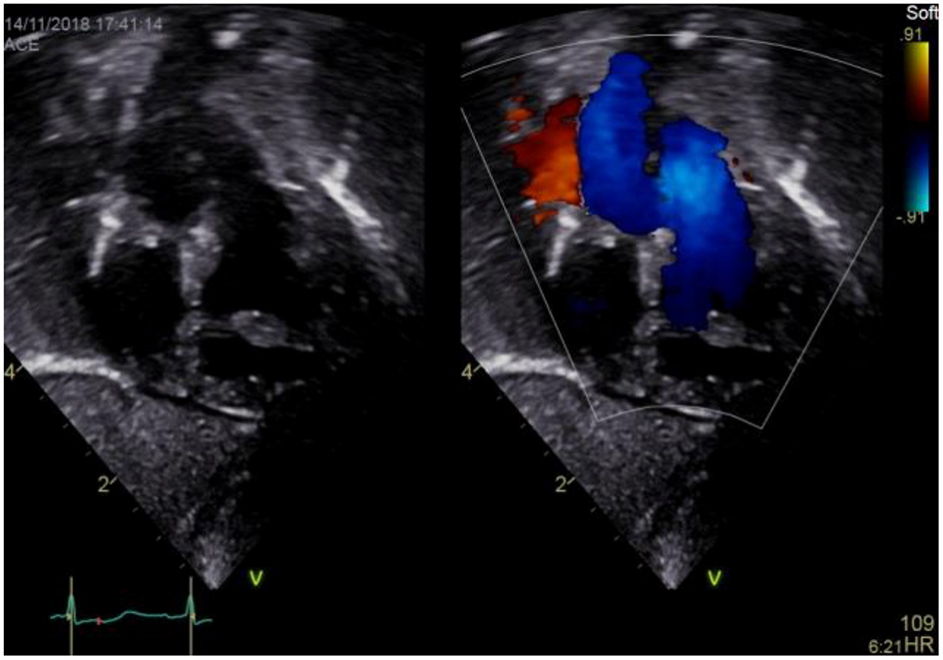
Subxiphoid “Right heart view”.

a) How to obtain (Figure 7):

From the subxiphoid four chamber view, as described above, rotate the transducer counterclockwise until you visualize the tricuspid valve, RV, RVOT and PV including the main pulmonary artery (MPA). Transducer dot pointing at 2 o'clock.

b) What to identify:

RA, TV, RV, RVOT, PV, MPA, IVS, LV in part.

c) Interpretation:

This view is best obtained in neonates and young infants and used to assess the right heart structures. TR, inflow *via* the tricuspid valve, hypertrophy or dilation of the RV, obstruction or dilation of the RVOT, and PR or pulmonary stenosis (sub-/supra-/valvular) may be identified. Tilting a bit more cranially may allow for visualization of the main pulmonary artery and its branches and thus evaluation for PDA or LPA/RPA stenosis. This “right heart view” is of particular value when assessing newborns or infants for Ebstein anomaly, Tetralogy of Fallot, or hypertrophied RV and allows for simultaneous imaging of RV function and PA flow (4). Moreover, pulmonary artery velocity time integrals (PaVTI) may be measured using pulsed wave Doppler to estimate pulmonary blood flow and RV output. Pulmonary artery acceleration time (PAAT) should be calculated and has been shown to be useful for evaluation of children and adult patients with PH (5, 11).

### Subxiphoid Sagittal Short Axis RV/LV View

**Figure 8:**
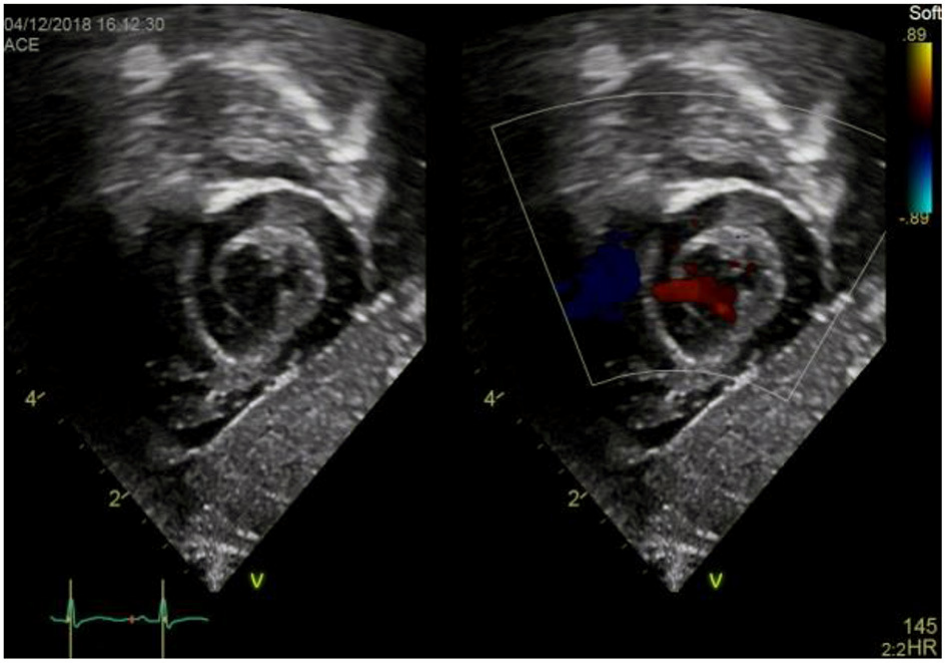
Subxiphoid short axis view.

a) How to obtain (Figure 8):

From the subcostal four chamber view, rotate the transducer clockwise until the heart is visualized in its short axis. Tilt the transducer, in order to make the view of the left ventricle as circular as possible. Usually the transducer dot points at 6 o'clock.

b) What to identify:

LV, RV, papillary muscles.

c) Interpretation:

This view allows for visualization of a round left ventricle in short axis. In case of elevated right ventricular pressure (e.g., pulmonary hypertension or pulmonary stenosis) the septal wall appears flattened and may form a D-shape (12). Muscular ventricular septal defects may be seen using color Doppler and RV size may be estimated. LV hypertrophy may be seen. Using plane area measurement, LV ejection fraction may be estimated using fractional shortening (FS) (13).

### Subxyphoid Sagittal Short Axis RVOT Sweep

**Figure 9:**
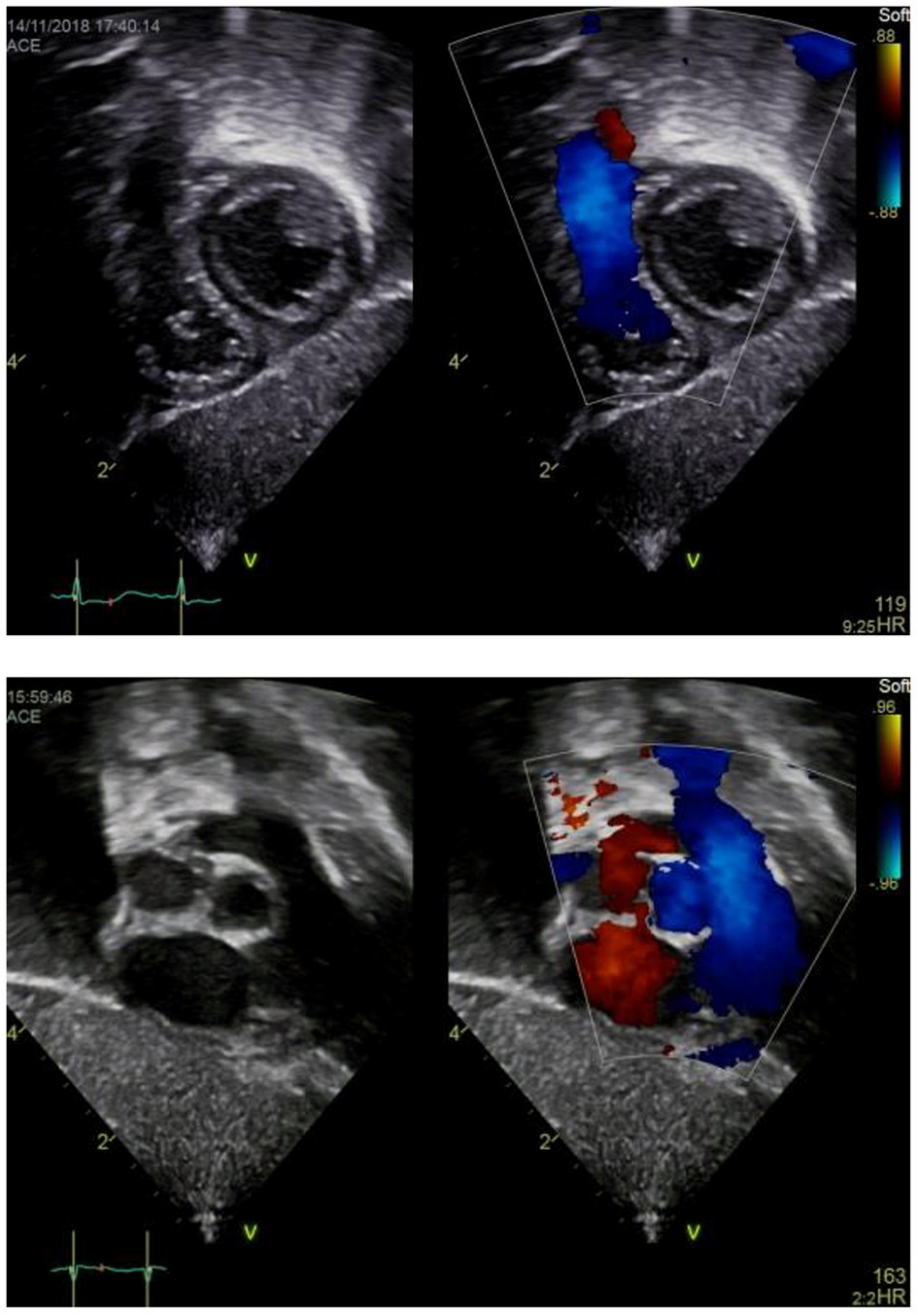
Subxiphoid short axis view–RVOT sweep.

a) How to obtain (Figure 9):

From the subxiphoid short axis view as described above tilt the transducer toward the patients' right side until you visualize the TV opening and closing within the RV and the right ventricular outflow tract (RVOT) and pulmonary valve (PV).

b) What to identify:

TV, RV, RVOT, PV, LV, MV in part.

c) Interpretation:

Pulmonary valve (PV) regurgitation may be seen as well as turbulent inflow *via* the tricuspid valve (TV) in case of e.g., TV stenosis. Using color Doppler, a muscular ventricular septal defect (VSD), especially an LV to RVOT shunt, as well as subvalvular and valvular PV stenosis may be identified e.g., in Tetralogy of Fallot.

### Subxyphoid Sagittal Short Axis Bicaval Sweep

**Figure 10:**
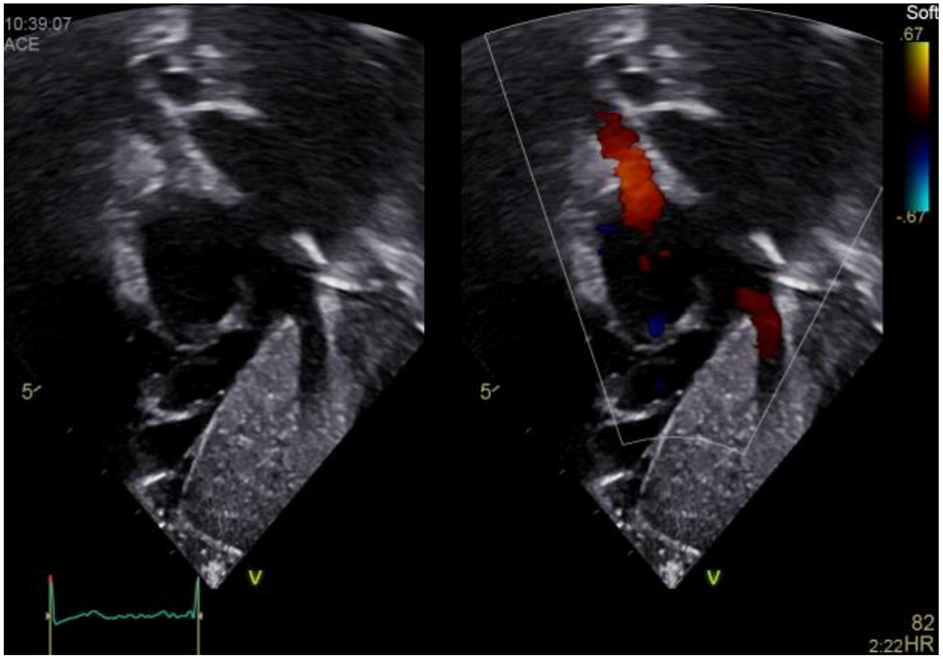
Subxiphoid short axis view–bicaval sweep.

a) How to obtain (Figure 10):

Tilt the transducer further to the patients' right until you visualize the IVS and superior vena cava (SVC) entering the RA.

b) What to identify:

RA, IVC, SVC, LA, IAS, right pulmonary artery (RPA).

c) Interpretation:

IVC and SVC inflow into the RA can be assessed and distinctive features of either of the central veins can be suspected by increased flow velocity using spectral Doppler. In particular, this view allows for evaluation of IVC/SVC inflow after atrial surgery involving patch closure of the IAS, which may lead to traction and aberration of the venous course. Furthermore, a PFO or ASD may be identified using color Doppler. Interruption of the IVC with azygous continuation can also be visualized from this view.

Subcostal window views intend to aid physicians performing FOCUS investigations. However, currently in children they cannot be recommended for exclusive use to determine cardiac anatomy and function due to lack of normative values in the pediatric cohort.

Outlining each the subcostal window views in detail above, we further provide a more comprehensive overview within the following tables (Tables 2, 3).

**Table 2:** List of available subcostal window views with assessable structures and functional parameters given for each view.

**Subcostal window view description**	**Assessment**
Subcostal abdominal short axis view (situs view)	Determination of normal abdominal visceral situs or otherwise situs anomalies, levocardia, pleural effusions, and movement of hemidiaphragms
Subcostal abdominal long axis view (IVC view)	IVC filling and collapsibility, hepatic vein distension or reverse flow, persistent foramen ovale (PFO) or atrial septal defect (ASD) using color Doppler
Sweep (Aorta)	Abdominal and descending aorta pulsatile flow pattern, diastolic flow, reverse flow pattern
Subcostal coronal long axis four chamber view	Pericardial effusions and both ASD and VSD, tricuspid or mitral regurgitation, left and right ventricular function using “eyeballing,” LV/RV size
Subcostal coronal long axis–Coronary sinus sweep	Prominent CS flow in PAPVR or TAPVR, CS ASD, persistent left superior vena cava
Subcostal coronal long axis–LVOT sweep	LVOT narrowing or LVOTO, aortic regurgitation (AR), aortic VTI
Subcostal coronal long axis–RVOT sweep or “right heart view”	Hypertrophy or dilation of the RV, obstruction or dilation of the RVOT, and PR or pulmonary stenosis (sub-/supra-/valvular), PDA or LPA/RPA stenosis; Ebstein anomaly, Tetralogy of Fallot
Subcostal sagittal short axis RV/LV view	D-shaping of LV, muscular VSD, RV size, LV hypertrophy, pericardial effusions, left ventricular function using “eyeballing”
Subcostal sagittal short axis RVOT sweep	PR, TV stenosis, muscular VSD–especially an LV to RVOT shunt, subvalvular and valvular PV stenosis
Subcostal sagittal short axis bicaval sweep	IVC and SVC inflow

*ASD, atrial septal defect; PFO, persistent foramen ovale; IVC, inferior vena cave; SVC, superior vena cava; LVOT, left ventricular outflow tract; LVOTO, left ventricular outflow tract obstruction; RVOT, right ventricular outflow tract; PR, pulmonary regurgitation; TV, tricuspid valve; PDA, persistent ductus arteriosus; VTI, velocity time integral; AR, aortic regurgitation; VSD, ventricular septal defect; LV, left ventricle; RV, right ventricle; LPA, left pulmonary artery; RPA, right pulmonary artery; PV, pulmonary valve; CS, coronary sinus; PAPVR, partial anomalous pulmonary venous return; TAPVR, total anomalous pulmonary venous return*.

**Table 3:** Structures and function parameters that may be assessed from the subxiphoid area views.

**Structure**	**Function**
Inferior and superior vena cava	Global left ventricular function
Hepatic veins	Global right ventricular function
Abdominal aorta	Aortic velocity time integrals
Diaphragm	Pulmonary artery velocity time integrals
Left and right atrium	Flow velocity *via* aortic valve
Left and right ventricle	Flow velocity *via* pulmonary valve
Atrial and ventricular septum	Shunt flow across the interatrial septum
Coronary sinus	Shunt flow across the interventricular septum
Pulmonary veins	Aortic regurgitation
Atrioventricular valves	Pulmonary regurgitation
Left ventricular papillary muscles	Position of central venous catheter tip
Aortic valve	Position of pacemaker leads
Pulmonary valve	Shunt *via* persistent ductus arteriosus
Ascending aorta	Dilation of cardiac chambers
Coronary arteries	Atrioventricular valve regurgitation
Main and branch pulmonary arteries	Intravascular filling
Pericardium	Estimation of central venous pressure
Right ventricular outflow tract	
Left ventricular outflow tract	
Ductus arteriosus	

## Conclusion

The use of FOCUS is well-established in pediatric emergency medicine, cirtical care and neonatal intensive care. Guidelines on the use of FOCUS exist but remain to be updated. As for all examiner-based techniques, education, and training to acquire and maintain a certain level of skill is of outmost importance, especially since FOCUS is usually performed by non-expert echocardiographers. Following a standardized protocol in order to assure quick and complete assessment of a limited number of diagnoses makes FOCUS highly valuable in clinical practice. Incorporation of subcostal window views into FOCUS may aid formulation of initial diagnosis and fast implementation of treatment. Since normative values for subcostal window views in pediatric patients, remain scarce, interpretation of findings from these views must be made with caution. It should be kept in mind that axis and angulation will differ to parasternal views, disabling the use of normative values derived from transthoracic views. Future research providing additional normative values and z-scores using a subcostal approach are required to improve the diagnostic value of subcostal window views.

## Author Contributions

SK-K and MK drafted the initial manuscript and reviewed and revised the manuscript. GH, HS, CT, and MC critically reviewed the manuscript for important intellectual content and revised the manuscript. CT recorded echocardiographic figures. All authors approved the final manuscript as submitted and agree to be accountable for all aspects of the work.

## Conflict of Interest

The authors declare that the research was conducted in the absence of any commercial or financial relationships that could be construed as a potential conflict of interest.
